# Relationship of frailty status with health resource use and healthcare costs in the population aged 65 and over in Catalonia

**DOI:** 10.1007/s10433-023-00769-8

**Published:** 2023-06-07

**Authors:** Àngel Lavado, Júlia Serra-Colomer, Mateu Serra-Prat, Emili Burdoy, Mateu Cabré

**Affiliations:** 1 Information Management Unit, Consorci Sanitari del Maresmes, Mataró, Barcelona Spain; 2 Clinical Research Unit, IMIM (Hospital del Mar Medical Research Institute), Barcelona, Spain; 3 Research Unit, Consorci Sanitari del Maresmes, Hospital de Mataró, Carretera de Cirera S/N, 08304 Mataró, Barcelona Spain; 4 CIBER-Liver and Digestive Diseases (CIBEREHD), ISCIII, Madrid, Spain; 5 Primary Care Department, Consorci Sanitari del Maresmes, Mataró, Barcelona Spain; 6 Internal Medicine Department, Consorci Sanitari del Maresmes, Mataró, Barcelona Spain

**Keywords:** Health resource use, Healthcare costs, Frailty, Population ageing, Hospitalizations, Primary care visits, Emergency visits, Outpatient visits

## Abstract

**Background:**

Frailty is a geriatric syndrome with repercussions on health, disability, and dependency.

**Objectives:**

To assess health resource use and costs attributable to frailty in the aged population.

**Methods:**

A population-based observational longitudinal study was performed, with follow-up from January 2018 to December 2019. Data were obtained retrospectively from computerized primary care and hospital medical records. The study population included all inhabitants aged ≥ 65 years ascribed to 3 primary care centres in Barcelona (Spain). Frailty status was established according to the Electronic Screening Index of Frailty. Health costs considered were hospitalizations, emergency visits, outpatient visits, day hospital sessions, and primary care visits. Cost analysis was performed from a public health financing perspective.

**Results:**

For 9315 included subjects (age 75.4 years, 56% women), frailty prevalence was 12.3%. Mean (SD) healthcare cost in the study period was €1420.19 for robust subjects, €2845.51 for pre-frail subjects, €4200.05 for frail subjects, and €5610.73 for very frail subjects. Independently of age and sex, frailty implies an additional healthcare cost of €1171 per person and year, i.e., 2.25-fold greater for frail compared to non-frail.

**Conclusions:**

Our findings underline the economic relevance of frailty in the aged population, with healthcare spending increasing as frailty increases.

## Introduction

Population ageing is a reality of concern in most developed countries. Frailty is a major and well-known clinical condition associated with ageing, characterized by a decrease in the body's functional reserves and in its ability to respond to external stressors (Morley et al. 2013). Because of the impaired functioning of various organs and systems, frail subjects are at increased risk of disease, adverse health outcomes, functional decline, falls, fractures, disability, and dependency (Lahousse et al. 2014). Prevalence of frailty in the population aged ≥ 65 years is about 11%, but greatly increases with age (Collard et al. 2012; Garcia-Garcia et al. 2011; Jürschik et al. 2012). In a context of an ageing population, frailty represents a huge potential public health burden (Ilinca and Calciolari 2015). For individuals, it implies a greater risk of adverse health outcomes, reduced autonomy, and decreased quality of life and, for society, it implies an increase in health and social resource use and the corresponding increase in expenditure. Ageing increases demand for healthcare services and costs (Alemayehu and Warner 2004; Dios-Guerra et al. 2021; Vela et al. 2019), and frailty has been associated with an increased use of primary care, hospital, and community services (Hoeck et al. 2012; Ilinca and Calciolari 2015). Several scientific literature has addressed the cost of frailty from different perspectives. Some studies have considered health care resource use and costs related to frailty in specific clinical conditions or in specific group of patients such as hospitalized patients with heart failure (Kwok et al. 2020), surgical patients (Eamer et al. 2019), or cardiac implanted patients (Patel et al. 2020; Mohamed et al. 2019). Other studies only considered costs of hospital care (Liotta et al. 2019; García-Nogueras et al. 2017) or only costs of ambulatory health care (Sirven and Rapp 2017) and others assessed the cost-effectiveness or cost-utility of interventions addressing frailty (Yokoyama et al. 2020; Li et al. 2020; Bleijenberg et al. 2017; Peña-Longobardo et al. 2021). Few studies evaluated cost of frailty in the community. They are from different countries such as USA (Ensrud et al. 2020; Johnston et al. 2020), China (Gao et al. 2021; Fan et al. 2021), England (Han et al. 2019), Germany (Hajek et al. 2018a, b) or Mexico (Salinas-Escudero et al. 2022), and despite the heterogeneity in their settings, designs, perspectives, cost elements considered, or applied rates, they all suggest an increase in health care expenditure according to frailty severity. A systematic review and meta-analysis about healthcare costs associated to frailty in community-dwelling aged population incorporating 5 original articles found a dose–response relationship between frailty severity and healthcare resource use and costs, but also conclude that further research is needed (Kojima 2019). What studies have been done confirm that frail subjects are the main consumers of health resources, that frailty greatly increases healthcare expenditure (Hajek et al. 2018a, b; Liotta et al. 2019; Martínez-Reig et al. 2018; Sirven and Rapp 2017), and also that healthcare costs are very context dependent. As far as we are aware, there is only one study in Spain assessing additional costs attributed to frailty (the FRADEA study) (García-Nogueras et al. 2017). As mentioned, this study only consider hospital costs but not ambulatory costs. To date, therefore, the cost of frailty in the Spanish setting is not well known. This study aims to assess hospital and ambulatory health resource use by general elderly population according to frailty status, and to estimate additional healthcare costs attributable to frailty.

## Material and methods

### Study design and population

A population-based observational retrospective cohort study was designed with follow-up from 1 January 2018 to 31 December 2019. Data were retrospectively obtained from computerized primary care and hospital medical records. The study population included all inhabitants aged ≥ 65 years ascribed to 3 primary care centres in the province of Barcelona (Catalonia, Spain). The study protocol was approved by the local ethical committee for clinical research.

### Study variables and data source

Frailty status was established according to the Electronic Screening Index of Frailty (e-SIF) (Serra-Prat et al. 2022), which includes the following clinical conditions: arthritis, atrial fibrillation, stroke, chronic renal failure, diabetes, heart failure, visual alterations, arterial hypertension, hypotension or syncope, coronary heart disease, dementia, osteoporosis or frailty fractures, Parkinson or neurodegenerative diseases, dyspepsia or gastroesophageal reflux disease, peripheral arterial disease, chronic lung disease, cutaneous ulcer, sleep disorders, inflammatory bowel disease or malabsorption, chronic liver disease, depression, sarcopenia, cachexia or muscular weakness, active cancer, psychosis, HIV infection, dysphagia, obesity, chronic pain, anaemia, weight loss in the last 6 months, anorexia or malnutrition, urinary or faecal incontinence, dyspnoea or fatigue, physical limitation or disability, dizziness or altered balance, falls in the last year, confinement or institutionalization, functional dependency or transfer problems, alcohol dependence, social vulnerability, polypharmacy, urgent admission in the last year with > 2 hospital days and age > 80 years. The e-SIF score is calculated by adding the clinical conditions present in a given time and is interpreted as follows: 0 to 4 as robust, 5 to 8 as pre-frail, 9 to 11 as frail and ≥ 12 as very frail. e-SIF was calculated for data corresponding to 1st of January 2018. Data were collected on institutionalization, planned and unplanned hospitalizations, major outpatient surgery hospitalizations, emergency visits, day hospital sessions, outpatient visits, and primary care visits, for both the study period (2018–2019) and the date of the event. The data used to calculate the e-SIF score were sourced from the primary care computerized medical history (e-CAP) for each subject, the pharmaceutical receipt database, and the hospital information system (HIS) of the reference hospital for the participating primary care centres. Data on age, sex, and health resource use during study period were obtained from e-CAP and HIS clinical records.

### Cost analysis

The cost analysis was performed from a public health financing perspective, in this case, for the Catalan Health Service (CatSalut). The cost elements considered were hospitalizations (planned, unplanned, and due to major outpatient surgery), emergency visits, day hospital sessions, outpatient visits, and primary care visits. Unit healthcare costs were obtained from public cost accounting contracts with CatSalut for primary and specialized acute care provision, published on the official CatSalut website (CatSalut). CatSalut establishes 2 charging methods: a unit cost per hospitalization, emergency visit, and day hospital session, and an annual budget based on morbidity, territorial and demographic factors for outpatient and primary care visits. Unit costs were obtained as follows: (a) for hospitalizations, the weighted average of the medical and surgical hospitalization costs according to real medical and surgical discharges in the year of study; (b) for emergency visits, the sum of the unit cost per visit and an additional established triage cost; (c) for day hospital sessions, as set out in the contract terms; (d) for outpatient visits, the annual budget divided by the real annual number of visits as obtained from the transparency portal annual report of the reference hospital (CSdM); and (e) for primary care visits, the annual budget of each primary care centre divided by the real annual number of visits, averaged for the 3 primary care centres participating in the study. Costing was as follows: €2147.82 per hospitalization, €210.0 per day hospital session, €107.71 per emergency visit, €56.42 per outpatient visit, and €19.59 per primary care visit. Finally, the total health cost was calculated as the sum of costs for hospitalizations, emergency visits, day hospital sessions, outpatient visits, and primary care visits.

### Statistical analysis

Frailty groups (robust, pre-frail, frail, and very frail) were compared for health resource use and health expenditure using the Kruskal–Walllis test (when considering all 4 frailty groups) and the Mann–Whitney *U* Test (when considering frail vs. non-frail categories). Percentage of attended subjects were compared using the Chi-square test. The effect of frailty on being attended to each of the health resources considered was evaluated using bivariate (unadjusted) and multivariate (adjusting for age and sex) logistic regression. The incremental cost of each frailty level in comparison to the previous frailty level was calculated using bivariate and multivariate lineal regression analysis (LRA), adjusting for age and sex. The incremental cost of frailty (frail vs. non-frail) was also calculated using bivariate and multivariate LRA. Statistical significance was established at *P* < 0.05.

## Results

A total of 9315 subjects were included in the analysis, mean (SD) age 75.4 (7.96) years, and 56% women. Frailty prevalence overall was 12.3%, with 52.8% of the study population considered as robust, 34.9% as pre-frail, 9.8% as frail and 2.5% as very frail. Frailty prevalence was 10.04% in men and 14.11% in women (*P* < 0.001) and increased progressively with age, rising from 3.2% for the 65–69 age bracket to 25.8% for the ≥ 95 age bracket (*P* < 0.001). Description of main characteristics of study population is presented in Table 1.

**Table 1:** Description of main characteristics of the study population

Clinical condition	Percentage	Clinical condition	Percentage
Arterial hypertension	52.56	Cancer	10.69
Polypharmacy	46.03	Dyspepsia	10.23
Arthritis	35.32	Social risk	9.82
Obesity	33.76	Chronic renal failure	7.64
Sleep disorders	21.56	Heart failure	3.95
Chronic lung disease	20.55	Dementia	3.32
Diabetes	19.29	Stroke	2.65
Depression	16.48	Neurodegenerative disease	0.98

Comparison of percentage of attended subjects between frail and non-frail and the unadjusted and adjusted effect of frailty on being attended at least once to each of the health resources considered is presented in Table 2. It shows a significant higher percentage of attended subjects in the frail group for all health resource considered, and an adjusted effect of frailty on all health resources considered except for institutionalization. Table 3 shows the average number of unplanned hospitalizations, planned hospitalizations, major outpatient surgeries, emergency visits, day hospital sessions, outpatient visits, and primary care visits per subject by frailty status, sex, and age for the period 2018–2019. Men used all services, except for primary care, more frequently than women. The rate of hospitalizations, emergency visits, and primary care visits for subjects aged > 80 years almost doubled that of subjects aged < 80 years. As frailty progressed, mean health resource use increased proportionally and significantly. Table 4 shows healthcare costs by frailty group. Higher frailty scores were associated with increased healthcare spending. Table 5 summarizes the bivariate and multivariate LRA results for the incremental cost attributed to frailty adjusted by age and sex. The progression from one frailty status to the next carries a €1392.21 increase in healthcare cost expenditure when adjusted by age and sex. Similarly, when considering only the frail versus non-frail categories, the multivariate LRA (adjusted for age and sex) shows an increased cost of €2342.58 attributable to frailty. The multivariate LRA also points to an independent effect of age and sex on healthcare costs. In the model that considers 4 frailty status categories, the interaction between age and sex did not reach statistical significance (*p* = 0.066), but in the model that considers frailty in two categories, a significant interaction was observed between age and sex (*p* = 0.034) on healthcare costs.

**Table 2:** Relationship between frailty and use of different health care resources (attended at least once)

	% of attended in non-frail	% of attended in frail	*p*	Unadjusted OR (95% CI)	Adjusted OR* (95% CI)
Unplanned hospitalization	12.86	38.82	< 0.001	4.30 (3.76–4.92)	3.20 (2.76–3.70)
Institutionalization	4.60	11.05	< 0.001	2.57 (2.08–3.18)	1.09 (0.87–1.37)
Emergency visit	45.79	74.67	< 0.001	3.49 (3.04–4.02)	2.88 (2.49–3.34)
Outpatient visit	64.46	86.07	< 0.001	3.41 (2.87–4.05)	3.62 (3.02–4.34)
Day hospital session	9.94	25.24	< 0.001	3.06 (2.63–3.56)	2.76 (2.35–3.25)
Primary care visit	93.41	98.96	< 0.001	6.68 (3.76–11.88)	7.42 (4.15–13.27)

*Adjusted by age and sex

**Table 3:** Healthcare resource use by frailty status, sex, and age group 2018–2019

	Unplanned hospitalizations		Planned hospitalizations		Major outpatient surgeries		Emergency visits		Outpatient visits		Primary care visits		Day hospital sessions	
	Mean (95% CI)	*P*	Mean (95% CI)	*P*	Mean (95% CI)	*P*	Mean (95% CI)	*P*	Mean (95% CI)	*P*	Mean (95% CI)	*P*	Mean (95% CI)	*P*
Robust	0.114 (0.10–0.13)	< 0.001	0.091 (0.08–0.10)	< 0.001	0.092 (0.08–0.10)	< 0.001	0.761 (0.72–0.80)	< 0.001	4.743 (4.50–5.00)	< 0.001	17.499 (17.03–17.97)	< 0.001	0.418 (0.32–0.51)	< 0.001
Pre-frail	0.304 (0.28–0.33)		0.180 (0.16–0.20)		0.149 (0.13–0.17)		1.365 (1.30–1.43)		8.564 (8.18–8.95)		33.670 (32.77–34.57)		0.932 (0.72–1.14)	
Frail	0.564 (0.50–0.63)		0.257 (0.21–0.31)		0.145 (0.12–0.17)		2.108 (1.94–2.27)		11.582 (10.67–12.49)		52.229 (49.81–54.65)		1.053 (0.82–1.29)	
Very frail	0.850 (0.70–1.10)		0.408 (0.28–0.53)		0.163 (0.10–0.23)		2.935 (2.53–3.34)		11.579 (9.78–13.38)		59.910 (55.28–64.53)		1.983 (1.17–2.79)	
Male	0.299 (0.27–0.32)	< 0.001	0.176 (0.16–0.19)	< 0.001	0.118 (0.10–0.13)	0.735	1.229 (1.17–1.29)	0.036	7.868 (7.50–8.23)	< 0.001	25.313 (25.53–27.09)	< 0.001	0.914 (0.75–1.08)	< 0.001
Female	0.200 (0.18–0.22)		0.123 (0.11–0.13)		0.121 (0.11–0.13)		1.103 (1.06–1.15)		6.184 (5.92–6.44)		28.622 (27.88–29.36)		0.532 (0.42–0.64)	
< 80 y	0.165 (0.15–0.18)	< 0.001	0.121 (0.11–0.13)	< 0.001	0.126 (0.12–0.14)	0.005	0.993 (0.95–1.03)	< 0.001	7.151 (6.88–7.42)	0.387	24.039 (23.51–24.57)	< 0.001	0.679 (0.57–0.78)	< 0.001
≥ 80 y	0.426 (0.39–0.46)		0.205 (0.18–0.23)		0.103 (0.09–0.12)		1.541 (1.46–1.62)		6.379 (6.02–6.74)		35.922 (34.66–37.18)		0.745 (0.55–0.94)	
Overall	0.243 (0.23–0.26)		0.146 (0.14–0.16)		0.119 (0.11–0.13)		1.158 (1.12–1.20)		6.919 (6.70–7.14)		27.612 (27.07–28.15)		0.699 (0.60–0.79)

**Table 4:** Healthcare costs (€) by frailty status, sex, and age 2018–2019

	Total cost		Unplanned hospitalizations		Planned hospitalizations		Major outpatient surgeries		Emergency visits		Outpatient visits		Primary care visits		Day hospital sessions	
	Mean (SD)	*P*	Mean (SD)	*P*	Mean (SD)	*P*	Mean (SD)	*P*	Mean (SD)	*P*	Mean (SD)	*P*	Mean (SD)	*P*	Mean (SD)	*P*
Robust	1420.19 (2523.3)	< 0.001	245.83 (993.6)	< 0.001	195.61 (827.0)	< 0.001	198.67 (769.3)	< 0.001	81.94 (150.3)	< 0.001	276.65 (493.4)	< 0.001	342.80 (328.9)	< 0.001	87.69 (721.8)	< 0.001
Pre-frail	2845.51 (3531.9)		653.54 (1609.8)		385.64 (1159.7)		320.82 (1027.3)		146.99 (205.5)		483.16 (631.6)		659.60 (509.9)		195.77 (1264.7)	
Frail	4200.05 (4275.8)		1212.25 (2083.7)		551.02 (1632.7)		311.86 (935.8)		227.06 (276.6)		653.45 (791.4)		1023.17 (731.9)		221.23 (767.6)	
Very frail	5610.73 (4823.0)		1825.19 (2473.7)		875.72 (2083.3)		350.29 (1108.6)		316.20 (334.1)		653.31 (786.7)		1173.63 (701.9)		416.39 (1319.8)	
Male	2555.93 (3762.8)	< 0.001	641.44 (1620.5)	< 0.001	378.31 (1299.6)	< 0.001	252.56 (886.1)	0.735	132.41 (218.2)	0.036	433.92 (667.6)	< 0.001	515.37 (495.9)	< 0.001	191.92 (1122.7)	< 0.001
Female	2093.33 (2977.5)		430.38 (1320.2)		263.88 (925.1)		258.97 (901.5)		118.81 (184.6)		348.88 (544.9)		560.70 (538.4)		111.72 (829.0)	
< 80 year	2008.57 (3122.7)	< 0.001	353.79 (1180.7)	< 0.001	259.82 (1019.9)	< 0.001	271.03 (918.2)	0.005	107.00 (185.0)	< 0.001	403.45 (623.1)	0.039	470.91 (425.9)	< 0.001	142.56 (901.1)	< 0.001
≥ 80 year	2961.81 (3745.2)		914.80 (1911.1)		439.38 (1274.4)		221.61 (837.0)		166.01 (226.1)		359.91 (553.6)		703.72 (645.0)		156.39 (1110.9)	
Overall	2295.20 (3350.6)		522.49 (1462.5)		313.81 (1105.6)		256.17 (894.8)		124.74 (200.1)		390.35 (603.4)		540.92 (520.7)		146.72 (968.9)

**Table 5:** Linear regression analysis (LRA) of the incremental cost attributed to frailty adjusted by age and sex

	Model 1: Frailty in 4 categories		Model 2: Frailty in 2 categories	
	Bivariate LRA (unadjusted)	Multivariate LRA (adjusted)	Bivariate LRA (unadjusted)	Multivariate LRA (adjusted)
Frailty	1400.097**	1392.214**	2499.181**	2342.583**
Age		184.5115**		546.1348**
Female		(−) 643.1798*		(−) 606.113**
Constant	1426.738**	1738.646**	1986.932**	2183.636**
*R*2	10.17%	11.09%	6.02%	7.23%

Beta coefficients expressed in €

^*^P < .05, **P < .001

**Fig. 1:**
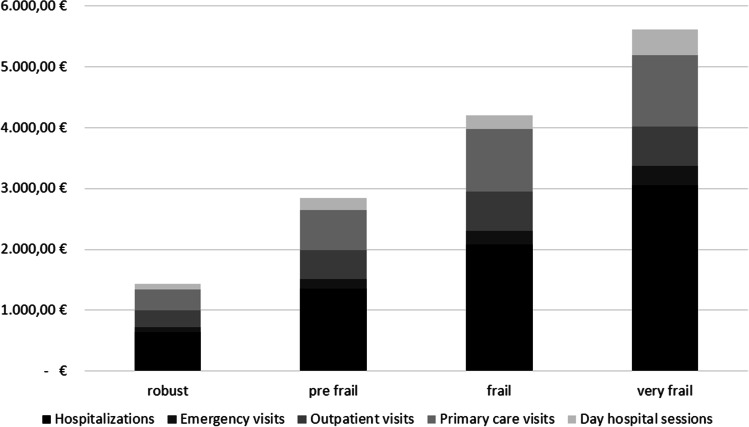
. Healthcare costs by frailty status

Figure 1 depicts total healthcare cost according to frailty groups. The hospitalization cost progressively contributed more to the total cost as frailty increased. While in the robust population hospital admissions represented 47.7% of the overall cost, this percentage raised to 58.7% in the very frail population.

## Discussion

Our findings confirm that frailty progression increases health resource use and that frailty increases healthcare costs by 125%, mainly due to hospitalizations. This represents an additional annual healthcare cost of approximately 1170€ per frail person compared to a non-frail person in our context. These results are similar to those reported by other authors in recent years. García-Nogueras et al. (2017) found, for the Albacete region of Spain, that pre-frail and frail patients cost €458 and €592 more, respectively, in total annual healthcare costs compared to robust patients. Sirven and Rapp (2017) showed, for France, that the incremental cost for ambulatory health expenditure was roughly €750 and €1500 for pre-frail and frail individuals, respectively. Although costs are context-specific and so may greatly vary between countries and healthcare financing systems, it can be generally agreed that healthcare costs are approximately 2.5-fold greater for frail individuals compared to non-frail individuals. Moreover, frailty has associated social costs due to dependency and disability that are not usually considered in these studies.

We found that older age groups were associated with higher frailty prevalence and with higher health resource use and cost, so age could act as a confounder when assessing cost of frailty. However, multivariate analysis showed an effect of frailty on healthcare costs independent from age and sex. Although frailty was more prevalent in women than in men, women used fewer health resources and ,consequently, had lower healthcare costs, with the multivariate LRA confirming the independent effect of sex on healthcare costs. Our results corroborate those of other studies conducted in the USA, India, and Spain (Cameron et al. 2010; Carretero et al. 2014; Mondal and Dubey 2020; Redondo-Sendino et al. 2006; Dalmau-Bueno et al. 2021), which have reported, adjusting by age and morbidity, lower health care expenditure and health care services use for women than men. We found, corroborating other Spanish studies (Aguado et al. 2012; Dios-Guerra et al. 2021), that the only service used more by women than men was primary care. Our findings are suggestive of gender inequality in health resource use. Causes of gender inequalities in healthcare use are complex and poorly understood. They may be related with socioeconomic status (women receive lower incomes) and social environment (twice as many women live alone as men), among others factors, and require to be further studied.

This study’s findings have 3 major implications for public policy. First, prevention-oriented interventions, mainly from the primary care setting, should play a key role in reducing the personal, social, and economic impact of frailty. Frailty, especially in its initial phases, is a preventable, reversible, and treatable condition (Cameron et al. 2013; Gill et al. 2006; Ng et al. 2015; Serra-Prat et al. 2017). More than a third of the population aged over > 65 year is classified as pre-frail, and are at an increased risk of becoming frail. Pre-frailty interventions for frailty prevention are both easier and more effective than frailty interventions to reverse this condition, so screening and intervention programmes for the pre-frail group would probably lead to greater reduction in health resource use and cost and would be more efficient than frailty treatments. Second, public health systems need to plan for and accommodate a growing demand for health resources by the elderly, as the prevalence of frailty in recent decades has been growing (Hoogendijk et al. 2021), and an important increase in the population aged > 65 years is forecast for the coming decades. In fact, even if frailty and pre-frailty prevalence could be reduced through preventive actions, the absolute number of frail and pre-frail subjects will increase due to population ageing. Healthcare institutions need to anticipate the increase in the frail population in order to coordinate health and social interventions that guarantee adequate care for the elderly. Finally, factors that affect access to health resources need to be considered a priority area for research, especially in the case of women. It has been suggested that widowhood could explain the gender gap in health resource use (Dios-Guerra et al. 2021; Mondal and Dubey 2020), as widowed women are more likely to experience economic deprivation. However, when access to public health is free, as in Spain, economic hardship cannot be the only explanation. Further research is needed to better understand healthcare use predictors for women, as the results should serve to design specific gender policies aimed at reversing inequality in access to health resources.

Main study limitations include: (a) the tariffs applied to translate health resource use into monetary units (€) are specific for our context, so results cannot be extrapolated to other settings or healthcare systems, (b) medication costs and social costs of institutionalization or care for dependency were not considered, c) the study considered only inhabitants ascribed to 3 primary care centres in Catalonia, and although they represent heterogeneous population segments (urban, suburban, and rural), the results cannot be extrapolated to the whole population, and d) although the e-SIF contemplates a large number of comorbidities, clinical conditions and polypharmacy and that the effect of frailty on healthcare costs has been adjusted for age and sex, we cannot rule out some residual confounding by other variables not included in the model and not part of the e-SIF. It is important to distinguish frailty from multi-comorbidity. While frailty is a geriatric syndrome characterized by greater vulnerability to suffering from illness and other adverse health outcomes, multi-comorbidity refers to the clinical condition of those people who accumulate two or more chronic diseases. Both concepts are closely related but must be distinguished because most but not all frail are comorbid and not all comorbid are frail. Despite this, with the definition of frailty according to the model of accumulation of deficits or clinical conditions, it is hard to differentiate which part of the increase in costs is due to frailty and which to comorbidity with demonstrated increased healthcare costs (Wang et al. 2018; Vela et al. 2019). This would require another operational definition of frailty that did not include comorbidities.

To sum up, in the population aged > 65, independently of age, as frailty increases, health resource use increases, to the point where the healthcare cost for frail subjects is more than double (2.25 times greater) that of non-frail subjects, representing an additional annual healthcare spend of nearly €1170 per frail person. For pre-frail subjects, the healthcare cost is also double that of robust subjects. Given that pre-frailty is much more prevalent than frailty in the population aged > 65, and that the effectiveness of preventive actions is high, interventions in the pre-frail group rather than in the frail group are likely to have a greater economic impact. Our results, which underline the economic implications of frailty in later life, suggest that postponing or reducing frailty will reduce healthcare costs and contribute to making the healthcare system more efficient and sustainable.

## References

[CR1] Aguado A, Rodríguez D, Flor F, Sicras A, Ruiz A, Prados-Torres A (2012). Distribution of primary care expenditure according to sex and age group: a retrospective analysis. Aten Primaria.

[CR2] Alemayehu B, Warner KE (2004). The lifetime distribution of health care costs. Health Serv Res.

[CR3] Bleijenberg N, Drubbel I, Neslo RE, Schuurmans MJ, Ten Dam VH, Numans ME (2017). Cost-effectiveness of a proactive primary care program for frail older people: a cluster-randomized controlled trial. J Am Med Dir Assoc.

[CR4] Cameron KA, Song J, Manheim LM, Dunlop DD (2010). Gender disparities in health and healthcare use among older adults. J Women’s Health.

[CR5] Cameron ID, Fairhall N, Langron C (2013). A multifactorial interdisciplinary intervention reduces frailty in older people: randomized trial. BMC Med.

[CR6] Carretero MT, Calderón-Larrañaga A, Poblador-Plou B, Prados-Torres A (2014). Primary health care use from the perspective of gender and morbidity burden. BMC Women’s Health.

[CR7] CatSalut. https://catsalut.gencat.cat/ca/coneix-catsalut/convenis-contractes/relacio/

[CR8] Collard RM, Boter H, Schoevers RA, Oude Voshaar RC (2012). Prevalence of frailty in community-dwelling older persons: a systematic review. J Am Geriatr Soc.

[CR9] CSDM. https://www.csdm.cat/ca/el-consorci/portal-de-transparencia/informacio-institucional/

[CR10] Dalmau-Bueno A, García-Altés A, Amblàs J, Contel JC, Santaeugènia S (2021). Determinants of the number of days people in the general population spent at home during end-of-life: results from a population-based cohort analysis. PLoS ONE.

[CR11] Dios-Guerra C, Carmona-Torres JM, Morales-Cané I, Rodríguez-Borrego MA, López-Soto PJ (2021). Evolution in the use of health services by older people in Spain (2009–2017). Health Soc Care Commun.

[CR12] Eamer GJ, Clement F, Holroyd-Leduc J, Wagg A, Padwal R, Khadaroo RG (2019). Frailty predicts increased costs in emergent general surgery patients: a prospective cohort cost analysis. Surgery.

[CR13] Ensrud KE, Kats AM, Schousboe JT, Taylor BC, Vo TN, Cawthon PM (2020). Frailty phenotype and healthcare costs and utilization in older men. J Am Geriatr Soc.

[CR14] Fan L, Hou XY, Liu Y, Chen S, Wang Q, Du W (2021). Catastrophic health expenditure associated with frailty in community-dwelling Chinese older adults: a prospective cohort analysis. Front Public Health.

[CR15] Gao K, Li BL, Yang L, Zhou D, Ding KX, Yan J (2021). Cardiometabolic diseases, frailty, and healthcare utilization and expenditure in community-dwelling Chinese older adults. Sci Rep.

[CR16] Garcia-Garcia FJ, Gutierrez Avila G, Alfaro-Acha A (2011). The prevalence of frailty syndrome in an older population from Spain. The Toledo study for healthy aging. J Nutr Health Aging.

[CR17] García-Nogueras I, Aranda-Reneo I, Peña-Longobardo LM, Oliva-Moreno JAP (2017). Use of healthcare resources and healthcare costs associated with frailty: the FRADEA study. J Nutr Health Aging.

[CR18] Gill TM, Gahbauer EA, Allore HG, Han L (2006). Transitions between frailty states among community-living older persons. Arch Intern Med.

[CR19] Hajek A, Bock JO, Saum KU, Matschinger H, Brenner H, Holleczek B (2018). Frailty and healthcare costs-longitudinal results of a prospective cohort study. Age Ageing.

[CR20] Hajek A, Bock JO, Saum KU (2018). Frailty and healthcare costs-longitudinal results of a prospective cohort study. Age Ageing.

[CR21] Han L, Clegg A, Doran T, Fraser L (2019). The impact of frailty on healthcare resource use: a longitudinal analysis using the Clinical Practice Research Datalink in England. Age Ageing.

[CR22] Hoeck S, François G, Geerts J, Van Der Heyden J, Vandewoude M, Van Hal G (2012). Health-care and home-care utilization among frail elderly persons in Belgium. Eur J Pub Health.

[CR23] Hoogendijk EO, Stolz E, Oude Voshaar RC, Deeg DJH, Huisman M, Jeuring HW (2021). Trends in frailty and its association with mortality: results from the longitudinal aging study Amsterdam, 1995–2016. Am J Epidemiol.

[CR24] Ilinca S, Calciolari S (2015). The patterns of health care utilization by elderly Europeans: frailty and its implications for health systems. Health Serv Res.

[CR25] Johnston KJ, Wen H, Joynt Maddox KE (2020). Relationship of a claims-based frailty index to annualized medicare costs: a cohort study. Ann Intern Med.

[CR26] Jürschik P, Nunin C, Botigué T, Escobar MA, Lavedán A, Viladrosa M (2012). Prevalence of frailty and factors associated with frailty in the elderly population of Lleida, Spain: the FRALLE survey. Arch Gerontol Geriatr.

[CR27] Kojima G (2019). Increased healthcare costs associated with frailty among community-dwelling older people: A systematic review and meta-analysis. Arch Gerontol Geriatr.

[CR28] Kwok CS, Zieroth S, Van Spall HGC, Helliwell T, Clarson L, Mohamed M (2020). The Hospital Frailty Risk Score and its association with in-hospital mortality, cost, length of stay and discharge location in patients with heart failure. Int J Cardiol.

[CR29] Lahousse L, Maes B, Ziere G (2014). Adverse outcomes of frailty in the elderly: the Rotterdam study. Eur J Epidemiol.

[CR30] Li Z, Habbous S, Thain J, Hall DE, Nagpal AD, Bagur R (2020). Cost-effectiveness analysis of frailty assessment in older patients undergoing coronary artery bypass grafting surgery. Can J Cardiol.

[CR31] Liotta G, Gilardi F, Orlando S (2019). Cost of hospital care for older adults according to their level of frailty. A cohort study in the Lazio region, Italy. PLoS ONE.

[CR32] Martínez-Reig M, Aranda-Reneo I, Peña-Longobardo LM (2018). Use of healthcare resources and healthcare costs associated with nutritional risk: the FRADEA study. Clin Nutr.

[CR33] Mohamed MO, Sharma PS, Volgman AS, Bhardwaj R, Kwok CS, Rashid M (2019). Prevalence, outcomes, and costs according to patient frailty status for 29 million cardiac electronic device implantations in the United States. Can J Cardiol.

[CR34] Mondal B, Dubey JD (2020). Gender discrimination in health-care expenditure: an analysis across the age-groups with special focus on the elderly. Soc Sci Med.

[CR35] Morley JE, Vellas B, van Kan GA (2013). Frailty consensus: a call to action. J Am Med Dir Assoc.

[CR36] Ng TP, Feng L, Nyunt MSZ (2015). Nutritional, physical, cognitive, and combination interventions and frailty reversal among older adults: a randomized controlled trial. Am J Med.

[CR37] Patel JN, Ahmad M, Kim M, Banga S, Asche C, Barzallo M (2020). Relation of frailty to cost for patients undergoing transcatheter aortic valve implantation. Am J Cardiol.

[CR38] Peña-Longobardo LM, Oliva-Moreno J, Zozaya N, Aranda-Reneo I, Trapero-Bertran M, Laosa O (2021). Economic evaluation of a multimodal intervention in pre-frail and frail older people with diabetes mellitus: the MID-FRAIL project. Expert Rev Pharmacoecon Outcomes Res.

[CR39] Redondo-Sendino Á, Guallar-Castillón P, Banegas JR, Rodríguez-Artalejo F (2006). Gender differences in the utilization of health-care services among the older adult population of Spain. BMC Public Health.

[CR40] Salinas-Escudero G, Carrillo-Vega MF, García-Peña C, Martínez-Valverde S, Jácome-Maldonado LD, Cesari M (2022). Last year of life, frailty, and out-of-pocket expenses in older adults: a secondary analysis of the Mexican health and aging study. J Appl Gerontol.

[CR41] Serra-Prat M, Sist X, Domenich R (2017). Effectiveness of an intervention to prevent frailty in pre-frail community-dwelling older people consulting in primary care: a randomised controlled trial. Age Ageing.

[CR42] Serra-Prat M, Lavado A, Cabré M, Burdoy E, Palomera E, Papiol M, Parera JM (2022). Development and validation of the electronic screening index of frailty (e-SIF). Age Ageing.

[CR43] Sirven N, Rapp T (2017). The cost of frailty in France. Eur J Health Econ.

[CR44] Vela E, Clèries M, Vella VA, Adroher C, García-Altés A (2019). Análisis poblacional del gasto en servicios sanitarios en Cataluña (España): ¿qué y quién consume más recursos? [Population-based analysis of the Healthcare expenditure in Catalonia (Spain): what and who consumes more resources?]. Gac Sanit.

[CR45] Wang L, Si L, Cocker F, Palmer AJ, Sanderson K (2018). A systematic review of cost-of-illness studies of multimorbidity. Appl Health Econ Health Policy.

[CR46] Yokoyama Y, Seino S, Mitsutake S, Nishi M, Murayama H, Narita M (2020). Effects of a multifactorial intervention for improving frailty on risk of long-term care insurance certification, death, and long-term care cost among community-dwelling older adults: a quasi-experimental study using propensity score matching. Nihon Koshu Eisei Zasshi.

